# Magnetic Helicity as a Predictor of the Solar Cycle

**DOI:** 10.1007/s11207-018-1332-3

**Published:** 2018-07-24

**Authors:** G. Hawkes, M. A. Berger

**Affiliations:** Centre for Geophysical and Astrophysical Fluid Dynamics, Exeter University, Exeter, Devon EX4 4PY UK

**Keywords:** Solar cycle, observations, Helicity, magnetic, Sunspots, statistics

## Abstract

It is well known that the polar magnetic field is at its maximum during solar minima, and that the behaviour during this time acts as a strong predictor of the strength of the following solar cycle. This relationship relies on the action of differential rotation (the Omega effect) on the poloidal field, which generates the toroidal flux observed in sunspots and active regions. We measure the helicity flux into both the northern and the southern hemispheres using a model that takes account of the Omega effect, which we apply to data sets covering a total of 60 years. We find that the helicity flux offers a strong prediction of solar activity up to five years in advance of the next solar cycle. We also hazard an early guess as to the strength of Solar Cycle 25, which we believe will be of similar amplitude and strength to Cycle 24.

## Introduction

In this article we investigate the suitability of the magnetic-helicity flux as a predictor of solar activity.

Solar activity and its associated phenomena and drivers are known to have wide-ranging effect on the heliosphere, including (for example) how cosmic rays pass through said regions (Ferreira and Potgieter, 2004). The Sun is the only star close enough for us to routinely observe magnetic activity regions with high spatial and temporal resolution. The magnetic field produced by the solar dynamo is utterly fundamental to furthering this understanding (Charbonneau, 2005; Cameron and Schüssler, 2015). Solar dynamos are in a rare class where we see a self-sustained and reinforced magnetic field (Moffatt, 1978). This and other conditions on the Sun (for example, sustained nuclear fusion) are not currently reproducible on the Earth, making it an excellent laboratory for studying more exotic and extreme physical phenomena.

There have been many attempts to make predictions of the solar-activity cycle, which itself tends to be quantified by either sunspot/active-region number or area. Prediction methodologies can be split into three subsets: extrapolation methods, precursor methods, and model-based predictions (Munoz-Jaramillo, Balmaceda, and Deluca, 2013). An extrapolation method would take advantage of, for example, a sunspot data series, and any mathematical relations that can be derived from them, whilst a precursor method takes advantage of other observables such as poloidal field strength during solar minima. Finally, a model-based prediction (arguably a combination of the two methods) takes a range of data sets in an attempt to model the solar cycle, which are then passed through evolution equations. A model-based prediction could include physical effects such as the $\alpha \Omega $ dynamo. Two recent reviews are Hathaway (2009) and Petrovay (2010).

One notable example is the work of Choudhuri, Chatterjee, and Jiang (2007), who use a mean-field dynamo model. In that article, they gave a prediction of the strength of Cycle 24, using a dynamo model-based prediction system, which has now been revealed to be accurate in at least an amplitude sense (a large reduction from Cycle 23). An earlier prediction for the same cycle, made by Dikpati, de Toma, and Gilman (2006), using a surface-flux-transport model, which had been successful in re-predicting previous solar cycles, predicted that Cycle 24 would in fact exceed Cycle 23 by a similar percentage to the decrease predicted by Choudhuri, Chatterjee, and Jiang (2007). This variability shows the difficulties in predicting solar cycles, even when using similar methods.

The work described in the current article fits within the definition of a precursor method, taking advantage of the magnetic-helicity flux during the preceding solar minima. We will attempt to display a relationship between this quantity and the strength of the following solar maxima, which is quantified here by sunspot number. We hope such a relationship would give us the ability to predict the strength of the cycle approximately five years in advance.

Magnetic helicity [$H$] quantifies how twisted a magnetic field is (Berger and Field, 1984). The general expression, for a magnetic field $\boldsymbol{B}$ within a magnetic surface $V_{\mathrm{M}}$ (*i.e.* $\boldsymbol{B}\cdot \hat{\boldsymbol{n}} = 0$) is given by

1 $$ H = \int _{V_{\mathrm{{M}}}} \boldsymbol{A} \cdot \boldsymbol{B} \, \mathrm {d}^{3} x, $$

where $\boldsymbol{A}$ is the vector potential of $\boldsymbol{B}$ (such that $\boldsymbol{B} = \nabla \times \boldsymbol{A}$). This expression is known to be gauge invariant. For a volume that does not fulfil the magnetic surface criteria, a gauge invariant relative helicity can be defined by

2 $$ H_{{{V}}} = \int _{{V}} (\boldsymbol{A} + \boldsymbol{A}_{P})\cdot (\boldsymbol{B} - \boldsymbol{P})\, \mathrm {d}^{3} x, $$

where $\boldsymbol{P}$ is typically a current-free field ($\nabla \times \boldsymbol{P} = 0$), where $\boldsymbol{P} = \nabla \times \boldsymbol{A}_{\mathrm{{P}}}$. $\boldsymbol{A}_{\mathrm{{P}}}$ is the vector potential of the current-free field. To ensure we are calculating the relative helicity of $\boldsymbol{B}$ only within $V$, we require $\boldsymbol{B} \cdot \hat{\boldsymbol{n}} = \boldsymbol{P} \cdot \hat{\boldsymbol{n}}$, where $\hat{\boldsymbol{n}}$ is the normal vector associated with $V$. On the Sun, the northern hemisphere generates and ejects negative helicity, and the southern hemisphere positive (Ding, Hong, and Wang, 1987).

Magnetic helicity is a well-conserved quantity in high-resistivity magnetohydrodynamics as a consequence of Alfvén’s frozen-flux theorem (Taylor, 1974). Berger and Field (1984) have also shown that helicity is still approximately conserved in less ideal conditions. This conservation means that helicity change with respect to time is essentially restricted to two mechanisms: helicity flow through and across a boundary (neglecting dissipation). We can then write (Berger and Ruzmaikin, 2000)

3 $$ \frac{\mathrm{d}H_{\mathcal{V}}}{\mathrm{d}t} = 2\oint _{ \mathcal{V}} (\boldsymbol{A}_{P} \cdot \boldsymbol{v}) B _{n} \,\mathrm {d}^{2} x - 2 \oint (\boldsymbol{A}_{P} \cdot \boldsymbol{B})v_{n} \,\mathrm{d}^{2} x, $$

where $\boldsymbol{v}$ is the fluid velocity flow on the solar surface. The first integral describes the flow of helicity due to tangential surface motions such as differential rotation. The second component of this integral describes helicity rising through the convective zone and being ejected into the corona, with a velocity component in the direction of $\hat{\boldsymbol{n}}$. In this study we choose to neglect the second component, and we focus on the contribution due to the Omega effect, which is the main contributor to large-scale helicity flux.

The Omega effect is typically attributed to the polar magnetic-field lines, which move slower than those at the Equator due to differential rotation. As a consequence, they wrap themselves around the solar axis, generating toroidal field. Thus, when we measure the strength of the polar field at solar minimum, we should get an estimate of how much toroidal flux is being stored for the production of sunspots in the next solar cycle. However, polar field (in this article and similarly in others) is defined as a field average over a 15-degree polar cap, whilst our helicity flux takes account of all of the field lines in a hemisphere. We believe that this makes helicity flux a better measure of the Omega effect than polar field.

Our model of the helicity flux is a three-stage process, tracing the flow of positive helicity. Negative helicity flux is ejected by the northern hemisphere into the corona, or equivalently is interpreted as positive helicity flowing into the interior. Positive helicity then flows through an equatorial slice into the southern hemisphere at a rate exceeding that of the inflow, resulting in a negative helicity build-up in the northern hemisphere. Equivalently, a small (relatively) flow of positive helicity exits the southern interior, which gives an overall build-up of positive helicity in the southern hemisphere. This model can be succinctly described by the following formulae (Berger and Ruzmaikin, 2000):

4 $$\begin{aligned} \frac{\mathrm{d}H_{{V\mathrm{N}}}}{\mathrm{d}t} =& \dot{H} (C_{\mathrm{ {N}}} \rightarrow V_{\mathrm{{N)}}}- \dot{H} (V_{\mathrm{{N}}} \rightarrow V_{\mathrm{{S}}}), \qquad \frac{\mathrm{d}H_{{C\mathrm{N}}}}{\mathrm{d}t} = - \dot{H}(C_{\mathrm{{N}}} \rightarrow V_{\mathrm{{N}}}), \end{aligned}$$

5 $$\begin{aligned} \frac{\mathrm{d}H_{{V\mathrm{S}}}}{\mathrm{d}t} =& \dot{H} (V_{\mathrm{ {N}}}\rightarrow V_{\mathrm{{S}}}) - \dot{H} (V_{\mathrm{{S}}} \rightarrow C_{\mathrm{{S}}}), \qquad \frac{\mathrm{d}H_{{C\mathrm{S}}}}{\mathrm{d}t} = \dot{H}(V_{\mathrm{{S}}} \rightarrow C_{\mathrm{{S}}}), \end{aligned}$$

where $V$ and $C$ denote the solar interior and corona, respectively, with $\mathrm{N}$ and $\mathrm{S}$ denoting North and South. In this article, when we refer to a sum of hemispheres, we will sum the absolute values of these quantities. The helicity, having built up within the hemispheres, can be ejected through CMEs, active regions, and *etc.* (van Driel-Gesztelyi, Démoulin, and Mandrini, 2003).

There have been studies into the effectiveness of measuring the build-up of magnetic helicity to measure the likelihood of a solar eruption (see Pariat *et al.*, 2017). This is admittedly restricted to singular events, rather than a study of helicity flow and build-up over the whole Sun. However, one advantage of this type of study is that one can take account of the scale of the active region, rather than assuming uniformity.

Sunspots will typically appear in pairs: regions of intense, oppositely signed magnetic toroidal flux. These are often referred to as bipolar magnetic regions (BMRs). This magnetic flux rises up from the convection zone via a process known as magnetic buoyancy (Parker, 1955). Sunspots are generated in their highest number around an equatorial band – where the toroidal field is assumed to be strongest (a consequence of meridional flow) (Mordvinov, Grigoryev, and Peshcherov, 2012). Sunspots will typically appear as dark spots on the photosphere, with temperatures between 3000 – 4500 K, in contrast with the surrounding material at a temperature of ${\approx}\, 5700~\mbox{K}$. This is due to an increase in magnetic pressure from the intense toroidal flux penetrating the surface, which limits convection. This effect can be observed in the values of the plasma $\beta $. Given their relation to the toroidal field (Balogh, Hudson, and Kristof, 2016), sunspots are an ideal measure of the Sun’s activity.

The Sun’s activity minimum is defined as the period in which we see very few sunspots, in between long periods of notable activity. At this time the Sun’s poloidal field is observed to be maximal. The action of differential rotation on the poloidal field (the Omega effect) then causes a maximal helicity flux in line with maximum poloidal field. The poloidal field that has been “wrapped up” by differential rotation goes on to be the toroidal flux rising out sunspots (the Babcock–Leighton mechanism).

Observations of the Sun have been performed for hundreds of years. Sunspot records are semi-reliably available back to 1610 (Hoyt and Schatten, 1998), although there is evidence of observations being made as early as the year 939 (Vaquero and Gallego, 2002). Other activity indices include the solar radio flux index (F_10.7_), interplanetary magnetic field (IMF), flare index, polar faculae (Sheeley, 2008), and coronal index (Fe xiv emission) (Usoskin, 2005).

There are multiple repositories containing some subset of these indices over a variety of time periods. The data used in this work are provided by the Wilcox Solar Observatory. Equally comprehensive is the Space Weather Prediction Centre (National Oceanic and Atmospheric Administration – NOAA), also offering up-to-date measurements alongside historical archives. Finally, the Kislovodsk Mountain Astronomical Station offers a large collection of data over a large time period – some of which is employed in this work.

In this paper, we analyse the data provided by the Wilcox Solar Observatory using a variety of techniques described within. This includes hemispherical splitting, and a direct comparison with the prediction capabilities of the polar field, which is currently the most commonly used precursor indicator of the solar cycle. We also test the strength of the inverse of our hypothesis: that sunspot number can predict a magnetic-helicity flux cycle. We also analyse some reconstructed magnetic-field data (Makarov and Tlatov, 2000), to which we apply the same techniques described in section one.

## Data and Analysis Techniques

**Figure 1:**
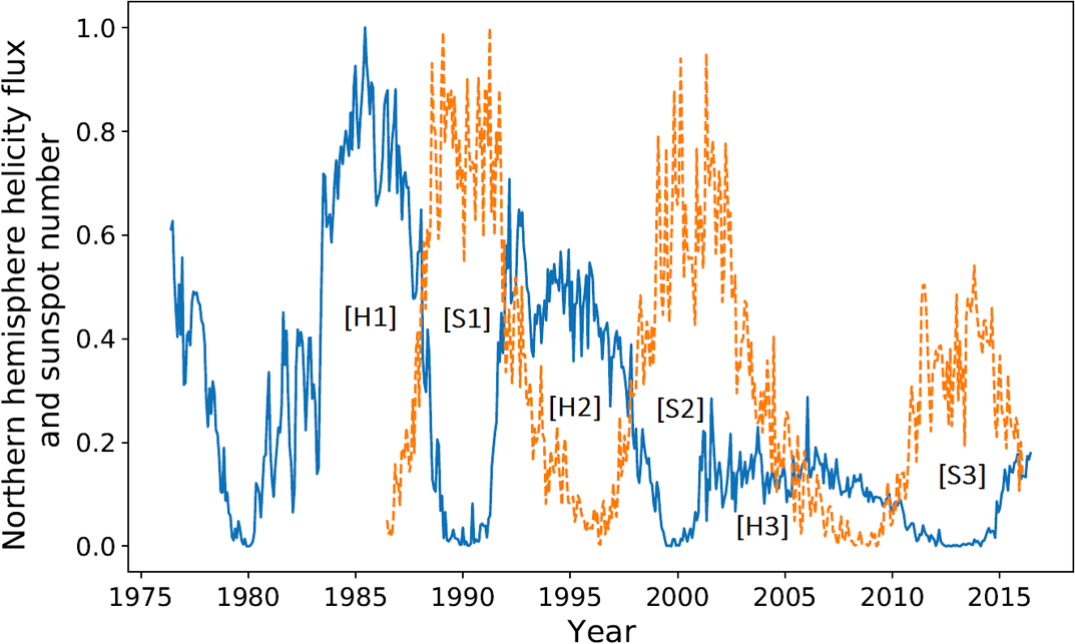
Sunspot number (*dashed orange*) and helicity flux through the northern hemisphere (*blue*), normalised by the amplitude of the largest cycle within the relevant data. Cycles have been labelled according to the number system used in this article, with “H” denoting helicity and “S” denoting sunspot.

In Figure 1, we compare the magnetic-helicity flux due to differential rotation through the northern hemisphere against the total sunspot number, averaged over each Carrington Rotation (CR). We take sunspot number over the entire solar disc due to restrictions in data availability. In later sections we do analyse the two hemispheres separately. Magnetic helicity is calculated using data from the Wilcox Solar Observatory, and sunspot data are provided by the WDC-SILSO, Royal Observatory of Belgium, Brussels. The helicities of the two hemispheres are not exact sign opposites, but they are aligned closely enough so that comparing a single hemisphere’s helicity to sunspot number over the disc should make little difference. Throughout this article, we normalise all data with respect to the highest peak contained within it, starting from the first relevant cycle. This would mean that we would not include, for example, a sunspot cycle before our first helicity cycle. It is possible to normalise these quantities using other techniques (such as taking $\mathrm{d}H \mathbin{/} \mathrm{d}t \, \Phi ^{-2}$, where $\Phi $ is the magnetic flux through a hemisphere): however, these methods were found to be less reliable and intuitive.

The data show a resemblance between the helicity cycles during solar decline/minima and the following solar-activity cycle. If we take account of the relationship between helicity flux and magnetic flux (${\sim}\, \Phi ^{2}$) *versus* that between sunspot number and magnetic flux (${\sim}\, \Phi $), the differences in amplitude are approximately accounted for. The similarities seem to be much weaker for the weaker cycle (the third pair of peaks (Cycle 24), 2000 onwards). This cycle is also distinctive in that the helicity maximum lies at around $20\%$ of that of the previous cycle. The cause of this anomaly could be the recent extended solar minimum (Frohlich, 2013). Regardless, both cycles are anomalously low within their own set, which is an inherent similarity.

The conclusion drawn is that a larger set of data is required. Magnetic-field data, however, are largely unavailable before the dates already graphed. We perform an analysis on the available data, with this restriction in mind.

### Dynamic Linear Modelling and Kalman Smoothing

Both data sets of Figure 1 have high frequency noise, making it difficult to identify similar overall trends. In an attempt to smooth the data, we employ two data-analysis tools: dynamic linear modelling (DLM) and Kalman smoothing (KS). Smoothed data are commonly used when working with sunspot number, for the purposes of prediction (Petrovay, 2010).

A dynamic linear model is described by two equations:

6 $$ y_{t} = F_{t} \theta _{t} + a_{t} \quad (a), \qquad x_{t} = G_{t} \theta _{t-1} + \omega _{t} \quad (b), $$

where $G_{t}$ and $F_{t}$ are matrices, $a_{t}$ and $\omega _{t}$ are vectors of Gaussian (normal) distributions (indexed by $t$), $y_{t}$ are the observations, and $x_{t}$ is the (assumed) underlying model. Equation 6a is known as the observation equation, and 6b is known as the state equation (Petris, Petrone, and Campagnoli, 2009). This state-space model assumes that the process (in this case the flux of magnetic helicity) is governed by some underlying process that we cannot measure directly (the state equation), but has an output that we can measure (the observation equation). This type of model is typically used to predict future data points, within an error given by the aforementioned Gaussian distributions. The underlying process (which is assumed to govern our observations) is found using maximum-likelihood estimation, based on the observations inputted (the data). In simple terms, we find the variables that are *most likely* to generate the data set that we observe, with associated distributions.

**Figure 2:**
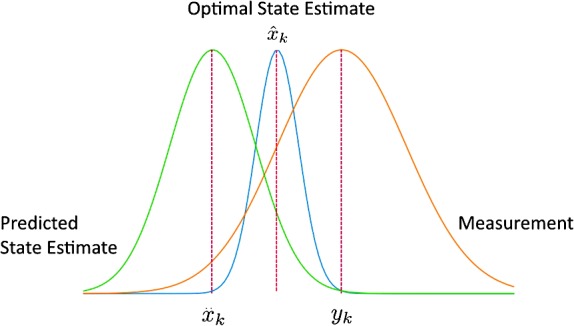
Pictorial representation of the Kalman smoothing process at an individual time step $t = k$. The optimal state estimate, in *blue*, is found by multiplying the *red* and *green curves* together, which represent our predicted state estimate and measurement distributions respectively.

Figure 2 shows how this model works, for an individual time step $t = k$. The optimal state estimate, shown by the grey curve, is calculated by multiplying the two distributions together, and it is assumed to be more accurate than either the measurement or state estimator. Calculating these optimal values for every time step $t$, once the entire data set is known, is referred to as Kalman smoothing (KS).

**Figure 3:**
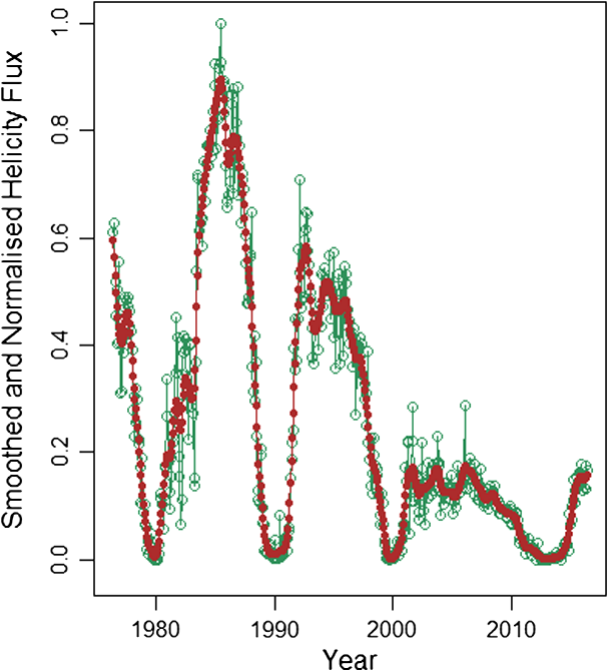
Normalised helicity data processed using Kalman smoothing and dynamic linear modelling.

**Figure 4:**
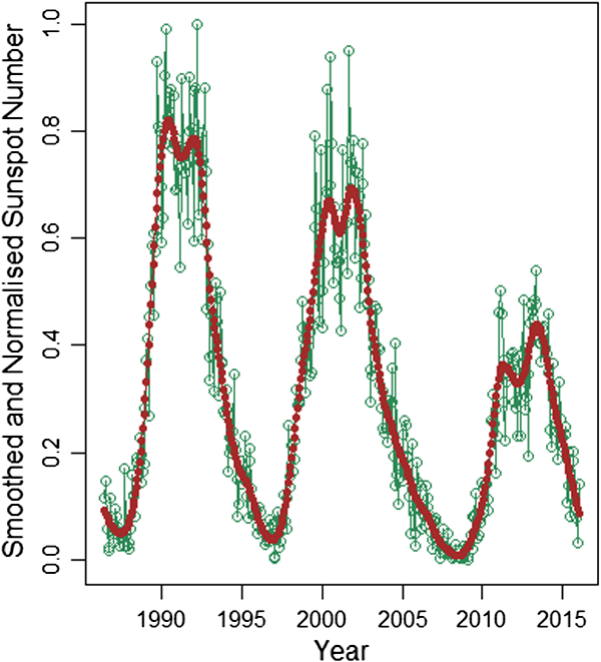
Normalised monthly sunspot data processed using Kalman smoothing and dynamic linear modelling.

In Figures 3 and 4, we give the data produced by applying the KS to the magnetic-helicity flux and the sunspot number, respectively. The red line indicates the smoothed data, and the green bars represent a $90\%$ confidence interval.

**Figure 5:**
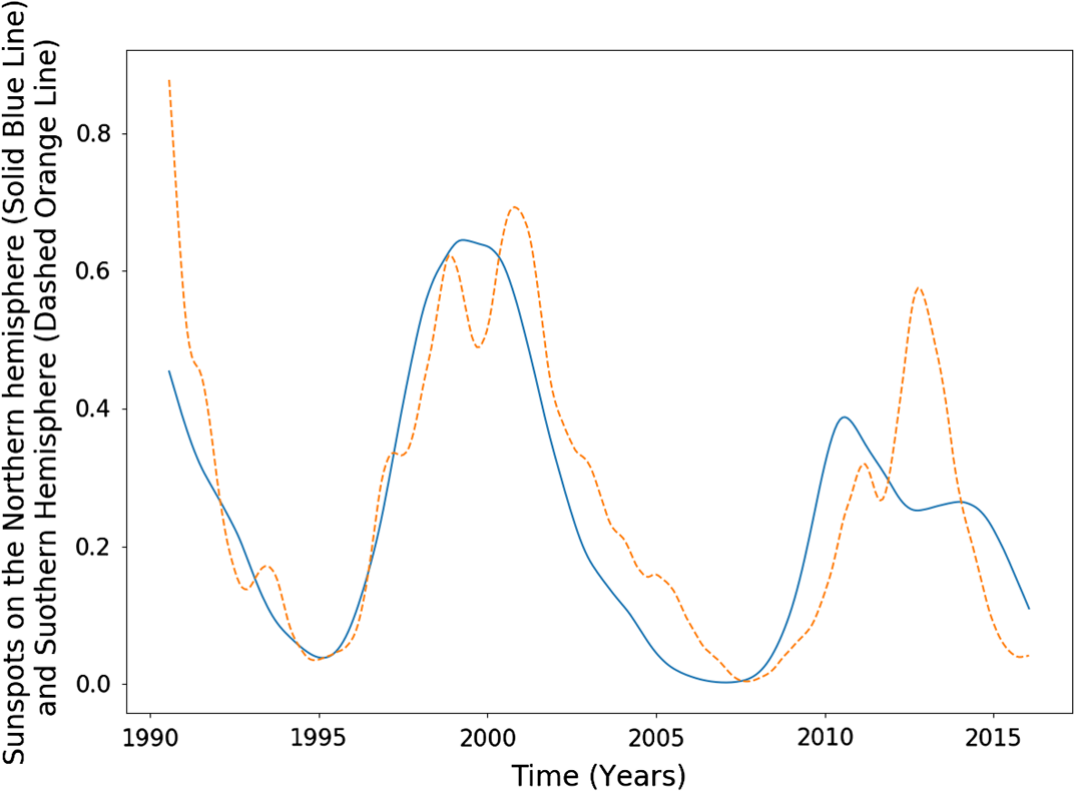
Smoothed sunspot numbers for the northern (*blue*) and southern (*dashed orange*) hemispheres from 1992 – 2017.

The smoothed plot of Figure 4 in particular reveals an interesting underlying double-peak structure, known as Gnevyshev gaps (Gnevyshev, 1967). It is likely this is due to a disparity in the specific timings of the dynamos governing each hemisphere. Evidence for this can be seen in Figure 5, where we have plotted sunspot number for the two hemispheres separately.

This two-peak structure is particularly notable in the final sunspot cycle, due to the disparity between the height of the two peaks, and the structures of the cycles.

### Pearson Correlation Coefficient

One standardized method for testing how well two data sets are correlated is the Pearson correlation coefficient (which we denote by $P$).

The discrete data form of the Pearson correlation coefficient is employed, given by

7 $$ P = \mathrm{corr}(X,Y) = \frac{\mathrm{cov}(X,Y)}{\sigma (X)\sigma (Y)}, $$

where $X$ and $Y$ are the two data sets being studied, $\mathrm{cov}(X,Y)$ is the covariance of the two sets, and $\sigma (X)$ and $\sigma (Y)$ are their standard deviations.

The correlation between the two data sets will be maximised at a given phase shift. We find that there is a clear difference between the optimum lag times, with the first (larger) peaks having a phase shift of 60 Carrington Rotations (4.5 years), whilst the second peaks are shifted by 92 Carrington rotations (6.9 years). These shifts give $P$-values for their relevant cycle pairs of 0.88 and 0.84 respectively, both of which indicate strong positive correlation. In all cases, unless otherwise stated, we use all data points that define the cycle to calculate correlation (between each successive minima).

**Figure 6:**
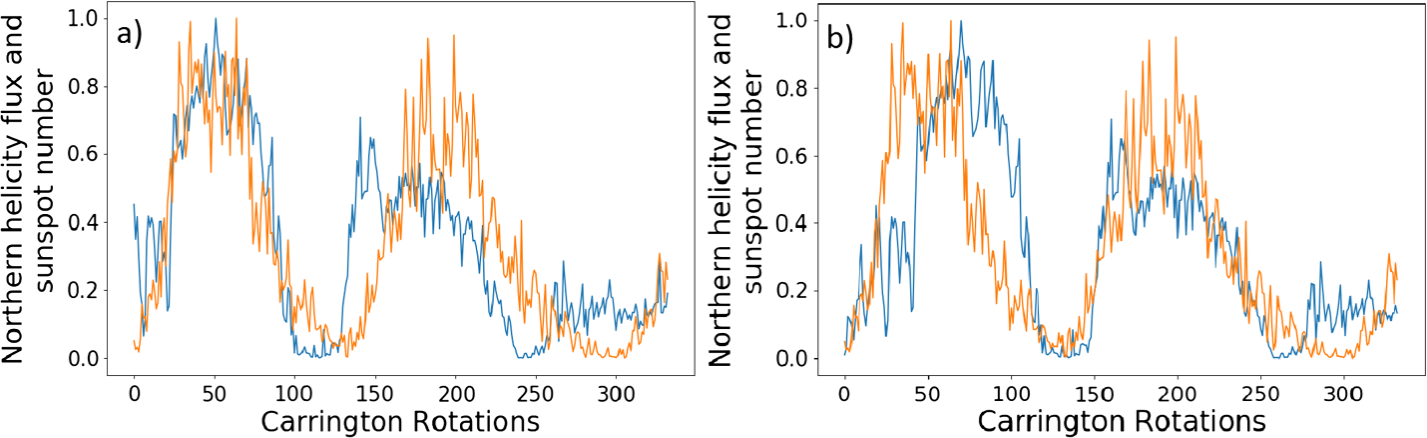
Phase-shifted normalised helicity data (*blue*) by 60 Carrington rotations (**a**) and 92 Carrington rotations (**b**), with normalised sunspot number (*orange*).

Figure 6 shows the helicity flux shifted by the above time periods plotted on the same axis as normalized sunspot number. Both phase shifts have their strong and weak points. For the latter, we see a good correlation of minima (lowest points between cycles), and excellent correlation for the second pair of peaks. The first pair of peaks is less well aligned (in the region between the minima). This is could be due to the sudden drop observed in the helicity flux around the fiftieth (CR 1692) Carrington rotation, which is not reflected in the sunspot relation. The 4.5 year phase shift gives a stronger correlation for the first pair of peaks, although the minima are no longer as well aligned. In the left figure, the second pair of peaks is less well aligned between the minima.

Note that the third pair of peaks is not very well correlated in either figure, nor do we attempt to perform an optimisation on those data sets. Figure 1 shows how distinct they are. We therefore do not perform an analysis on this peak for sunspot number over the entire disc.

**Figure 7:**
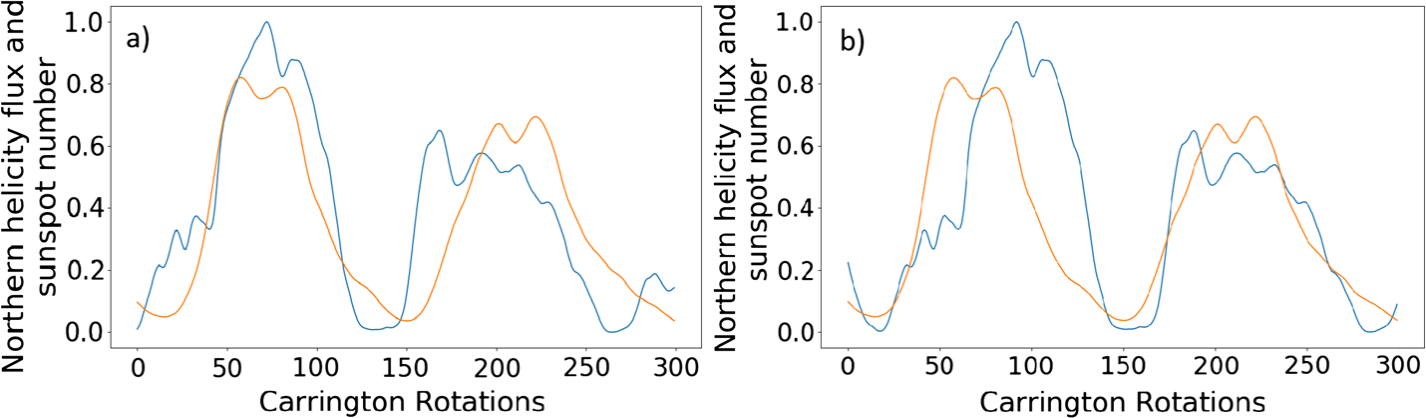
Phase-shifted smooth normalised helicity data (*blue*) by 98 Carrington rotations (**a**) and 68 Carrington rotations (**b**), with normalised smooth sunspot number (*orange*).

We also plot the smoothed versions of these graphs, with identical phase shifts, in Figure 7. The results of this are similar to that of the previous figure. Visually, the peaks appear less correlated. The values of $P$, however, are greater for the two optimised cycles. The left of Figure 7 corresponds to the phase shift of 60, and gives a $P$-value of 0.938 for the first cycles, and on the right we have a phase shift of 92 with $P = 0.904$ for the second pair of cycles. This is a marked improvement for the latter in particular. The comments made on Figure 6 regarding the structural differences continue to apply here.

### Integration

In the previous section we have noted that whilst some peaks of our data sets are well aligned, the Cycle 24 peak in particular offers only weak indications of correlation, both in terms of length and amplitude. We therefore look for a data-analysis technique that disregards structure, reducing large sets of data to singular points. Given that we are looking to compare measures of activity, we decided to compare the area under adjacent peaks. The reader should note that these areas have been calculated after the normalisation procedure, and therefore have units of Carrington rotations. The first example of this is shown in Table 1.

**Table 1:** Values of integrated helicity flow and sunspot number with helicity shifted forwards.

Peak pair	Helicity data integrated	Sunspot number integrated	Ratio of integrations
1	59.50 CR	55.15 CR	94%
2	42.39 CR	52.40 CR	81%
3	17.5 CR	26.50 CR	66%

Integrating helicity flux [$\mathrm{d}H / \mathrm{d}t$] over time clearly gives the total helicity [$H$] that passed through the corona in a given time period. For sunspot number, a temporal integral is less physically meaningful. However, we do note the relation between sunspot size, activity, and its period of existence (Henwood, Chapman, and Willis, 2010). For recurring sunspots (those that traverse the entire solar disc and re-appear in a following Carrington rotation), the temporal integral should take account of their increased size and activity.

The ratio column of Table 1 (and all subsequent tables) is obtained by dividing the smaller quantity by the larger, regardless of association. We see that, for the second peak in particular, the areas under the curves are very similar. The weakest comparison comes from the third pair of peaks. All three results match with a 66$\%$ threshold, but with an average of 80$\%$, indicating good area matching.

## Hemispherical Helicity and Sunspots

**Figure 8:**
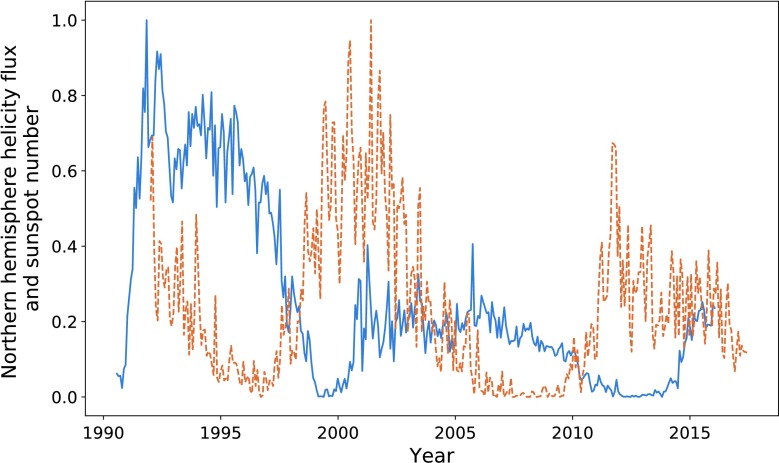
Northern hemisphere helicity flow (*blue*) and sunspot number (*dashed orange*).

**Figure 9:**
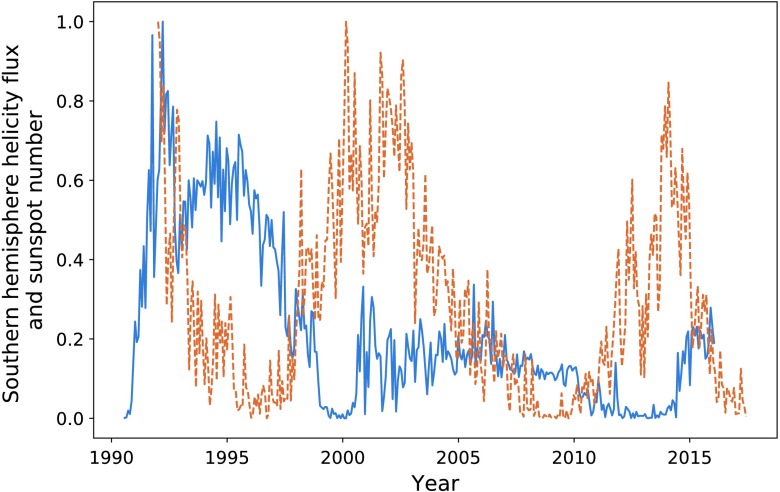
Southern hemisphere helicity flow (*blue*) and sunspot number (*dashed orange*).

In Figures 8 and 9 we plot the sunspot number for each hemisphere against the helicity flow for the respective hemisphere. Sunspot number that has already been split by hemisphere is only available from 1992 onwards, restricting the data analysis. The limited results do indicate a stronger relationship than that when the hemispheres are combined. In particular, if we look at the final two cycles of Figure 8, they appear to be more closely related than what we observe in Figure 1. The most recent cycle in particular is much closer in amplitude. This is, however, not reflected in the southern hemisphere (see Figure 9).

Table 2 shows the results of calculating the ratios of the areas for cycles when we split sunspot number by hemisphere. We see better results (on average) here than in Table 1, with an average ratio of $85.9\%$, indicating a strong correlation between the area enclosed by each cycle. Additionally, the final column shows the result of the integration ratio when we take the sum of absolute helicity flux from both hemispheres, giving results stronger than the above average.

**Table 2:** Ratios of the values of integrated helicity flow and sunspot number separated by hemisphere.

Peak pair	Integration ratio (North)	Integration ratio (South)	Ratios of sums
2	73.5%	98.7%	94.9%
3	93.2%	78.0%	83.1%

Maximising correlation for the first pair of peaks for each hemisphere gives $P = 0.76$ with a lag of 77 CR in the North, and $P = 0.72$ and a 88 CR lag in the South. We encounter a problem when attempting to map the second helicity cycle onto its sunspot counterpart, as the shifted helicity has a length exceeding the sunspot data range. Correlating the minima gave a lag of 94 CR for the northern hemisphere and 98 for the southern. This fits in with our pattern of varying lag time. The two values of $P$ obtained indicate strong positive correlation between the cycles.

**Figure 10:**
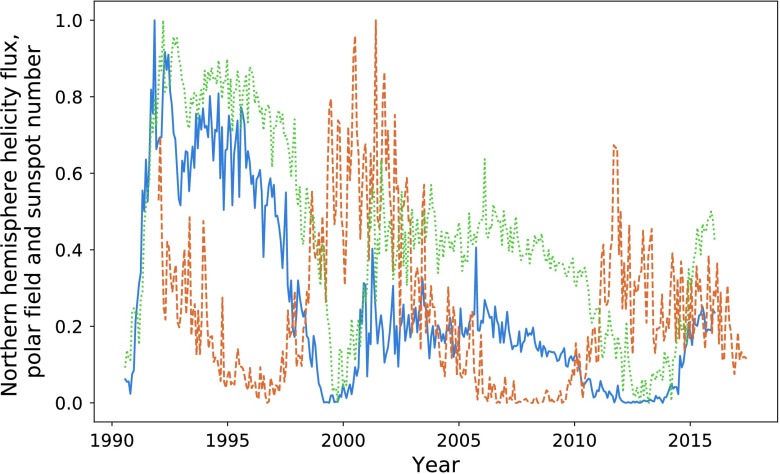
Northern hemisphere helicity flux (*blue*), sunspot number (*dashed orange*), and polar field (*dotted green*).

In Figure 10 and Table 3, we make comparisons with the predictively capabilities of the polar field, defined as the absolute value of the average radial field in the $0 \leq \theta \leq 15$ range for the northern pole, and similarly for the southern pole. Visually, the polar field is less well correlated with the sunspot number, and we see almost consistently lower integration ratios. The integration ratio for the second peak in the northern hemisphere is a relative anomaly. However, this unusually high value is still lower than that offered by the magnetic-helicity flux.

**Table 3:** Ratios of the values of integrated polar field and sunspot number separated by hemisphere.

Peak pair	Integration ratio (North)	Integration ratio (South)
2	54.8%	90.4%
3	41.1%	55%

### Summation of Hemispheres

**Figure 11:**
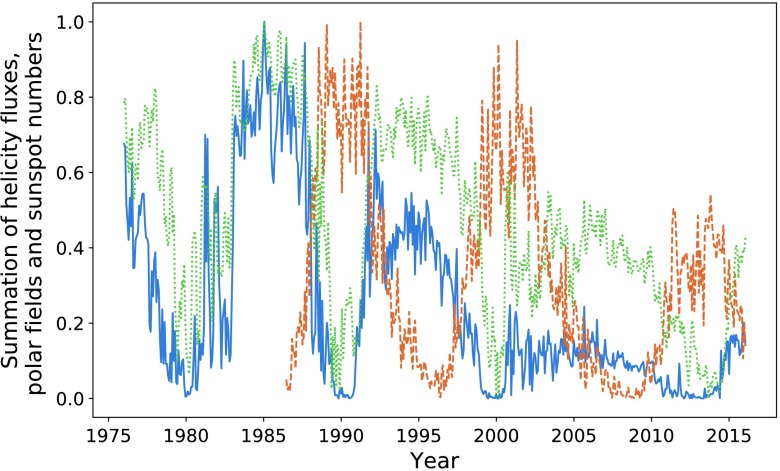
Helicity flux (*blue*), polar field (*dotted green*), and sunspot number (*dashed orange*).

In Table 2, we see that when using a summation over the two hemispheres, we obtain slightly improved integration ratios. In the previous section we have been working only with the northern hemisphere helicity flux plotted against sunspot number for the entire solar disc. Here we perform this same summation procedure, inclusive of the first cycle. Figure 11 gives the result of normalising the summation of the helicity fluxes and polar fields within both hemispheres. We obtain correlation values of $P = 0.86$ and $P = 0.83$ for the helicity cycles (when compared to sunspots cycles), and $P = 0.85$ and $P = 0.80$ for the polar fields, which is approximately equivalent to the result obtained when observing the northern hemisphere only. The results of integration analysis are given in Table 4. The polar field is seen to occasionally outperform the helicity flux as a precursive quantity only in terms of amplitude, and then only if we do not split sunspot number by hemisphere.

**Table 4:** Integrated helicity flux and polar field when using hemisphere summation.

Peak pair	Helicity integration ratio	Polar field integration ratio
1	90.0%	67.9%
2	75.4%	72.3%
3	58.4%	45.2%

### Sunspot Area

The limited extent of the hemispherical sunspot number data can be partially overcome with publicly available hemispherical sunspot area, which is recorded dating back to 1874, available from NASA (solarscience.msfc.nasa.gov/greenwch.shtml).

**Figure 12:**
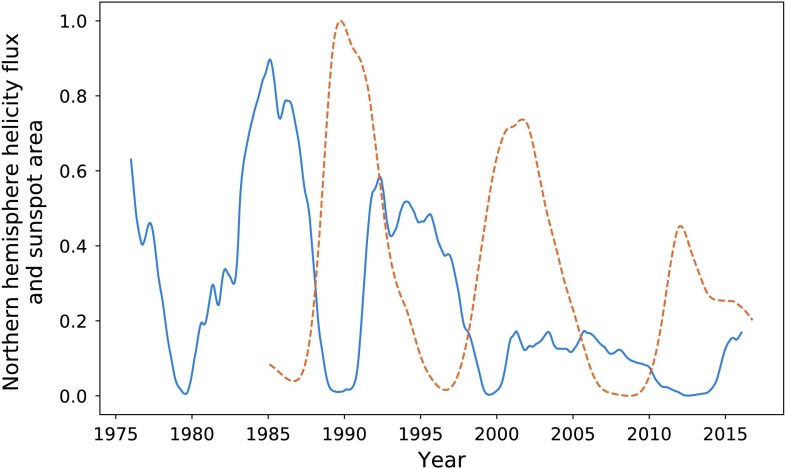
Helicity flux (*blue*) and sunspot area (*orange dashed*) in the northern hemisphere.

In Figure 12, we plot helicity flux against sunspot area for the northern hemisphere, from 1976 onwards. Sunspot-area data are highly noisy and have thus been smoothed and compared to the smoothed helicity flux. The first pair of cycles, for which we could not obtain “real” hemispherical sunspot number, gives an integration ratio of $88.8\%$. The visual correlations are approximately equivalent to that of Figure 10, except for a slight amplitude increase in the final sunspot cycle (although this could be an artefact of the smoothing process).

## Interpolated Sunspot Data

Temmer *et al.* (2006) have generated a catalogue of sunspot data, split by hemisphere, for the years 1945 – 2004. This was performed using drawings taken from two separate observatories, the results from which were normalised using the international sunspot number. More details can be found in their article.

**Figure 13:**
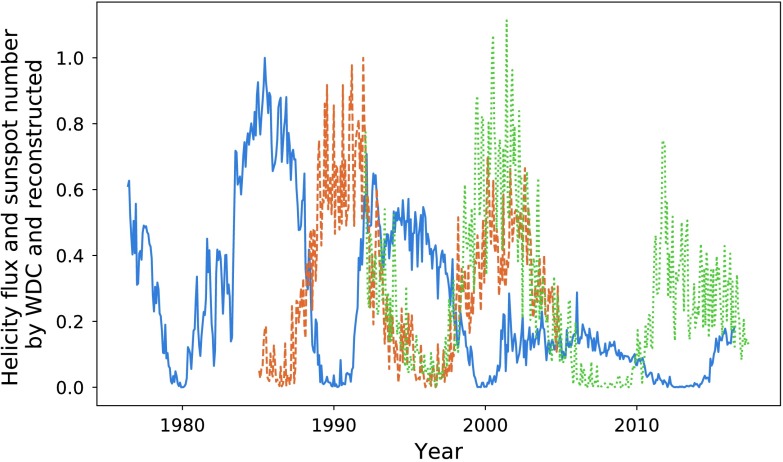
Helicity flow (*blue*), WDC sunspot number (*dashed orange*), and reconstructed sunspot number (*dotted green*).

Hemispherical data were calculated on a daily cadence, allowing us to calculate means over a Carrington rotation. Normalising this gives the data set shown in Figure 13. We have also included the observed WDC data as an indication of accuracy. We choose to apply this data only to our initial helicity cycle, given that this is the only cycle for which we do not have already existing hemispherical sunspot data. The normalisation procedure for the hemispherical sunspot data would thus be centred around the cycle encompassing 1985 – 1998.

Calculating the integration ratios for the North gives a value of $74\%$ for the North and $82\%$ for the South. These values have a median of $78\%$, which is considerably lower than that obtained when one uses sunspot number over the entire solar disk ($90\%$). This could be due to the reconstruction, given the improvements observed in the ratios when using the WDC data (especially in the case of the final cycle).

## Sunspots Predicting Helicity Flow

In order to assert statistically that it is indeed helicity predicting the behaviour of the sunspot cycle, and not the converse, we must test the strength of said converse. Therefore, in this section we look at the strength of the theory that sunspot number predicts helicity flow. We must note that by including an additional sunspot cycle, the normalisation of the sunspots is changed slightly.

**Figure 14:**
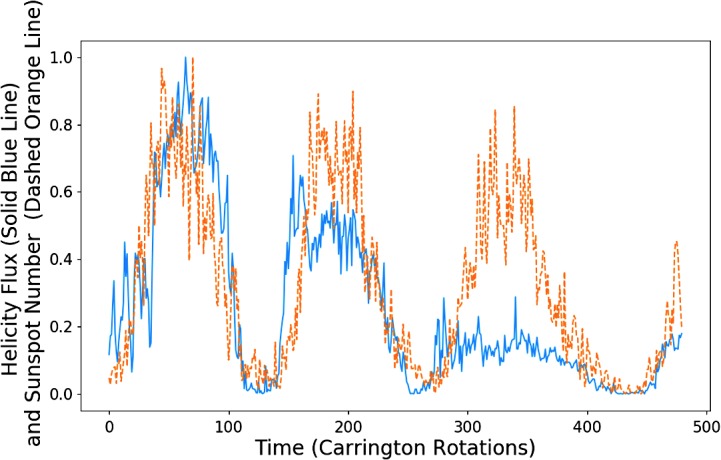
Helicity flow (*solid blue*) and sunspot number (*dashed orange*) shifted forward by approximately 3.33 years.

Figure 14 shows sunspot number on the same axes as helicity flow, where the sunspot number has been shifted forwards by approximately 4.2 years. This value was chosen using the same process of correlation maximisation described in the previous section.

We notice that the minima in particular are very well matched, as opposed to the changing phase shift of the helicity → sunspot figures. In particular, the minima of the final pair of peaks, where we saw an elongated helicity cycle, are well correlated.

We see very weak correlation for the final pair of peaks in terms of amplitude. This weakness is quantified in Table 5, where we have performed the same procedure as described for Table 1. The ratio is worse for the first pair of peaks ($- 4\%$), slightly improved for the second ($+ 6\%$), and drastically worse for the final pair ($-29\%$).

**Table 5:** Integrated helicity flux and sunspot number with sunspot number shifted forwards.

Peak pair	Helicity data integrated	Sunspot number integrated	Ratio of integrations
1	59.5 CR	53.50 CR	90.0%
2	42.39 CR	48.90 CR	87.2%
3	17.5 CR	46.79 CR	37.4%

We find that, excluding the final pair of peaks, a value of $P = 0.804$ is achieved.

One explanation for these results is that cycle influence works not only in one direction, but both. We appear to see the cycle amplitude/strength (where strength is indicated by the integrated area) of a sunspot cycle being dictated by helicity flow, whilst the length of a helicity flux cycle is strongly correlated with that of the previous activity cycle.

## Comparisons with Polar Field

The strength of the polar field during solar minima has often been used to predict the strength of the following solar maxima (Jiang, Chatterjee, and Choudhuri, 2007). The dipole moment has also been used (such as in the predictions made in Choudhuri, Chatterjee, and Jiang, 2007), back to 1978 (Schatten *et al.*, 1978). However, given that the polar field is more directly related to the Omega effect being described by our helicity flux, as well as a good measure of the dipole moment, we choose to compare the effectiveness of magnetic-helicity predictions with this benchmark. Helicity flux, as stated earlier, is a closer measure of the Omega effect than the polar field, making it likely to be more strongly correlated with sunspot number.

**Figure 15:**
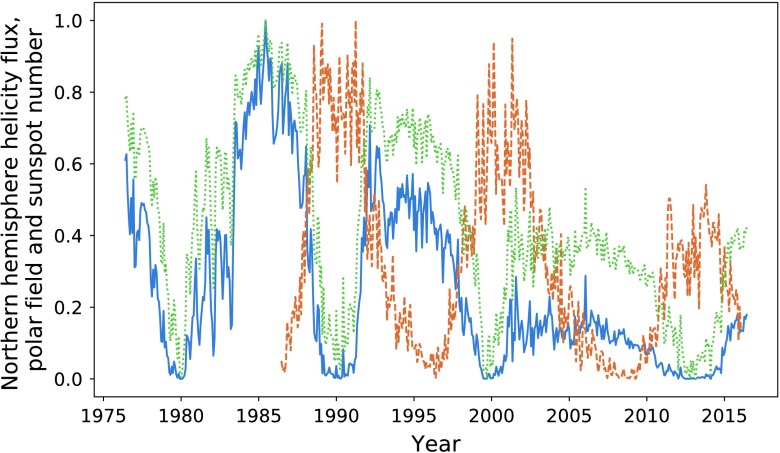
Normalised magnetic-helicity flux (*blue*) and polar field (*dotted green*) for the northern hemisphere compared with normalised sunspot number (*dashed orange*).

In Figure 15, we plot the magnetic-helicity flux through the northern hemisphere compared with total sunspot number and the averaged northern pole magnetic-field strength. We continue to define the northern pole as the region $0 \leq \theta \leq 15 ^{\circ }$, for which we take an absolute value of the average field. The southern pole is defined similarly. Visually, the polar field appears to be a slightly weaker precursor for the strength of the solar minima, except in the case of the final pair of peaks, where it performs much better than magnetic helicity.

Of note is the amplitude differences between the polar field and magnetic helicity. Given that the expression used to calculate the magnetic-helicity flux is directly dependent upon the poloidal field, we would expect a closer relation in amplitude. These differences are likely due to the distribution of flux being more concentrated at mid to low latitudes.

**Figure 16:**
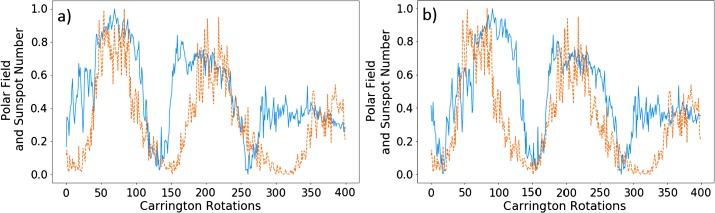
Normalised polar field (*blue*) with a phase shift of 60 CR (**a**) and 92 CR (**b**) plotted alongside sunspot number (*dashed orange*).

In Figure 16, we plot the polar field against sunspot number with optimised phase shifts. Here, we found that correlating the first pair of peaks required a phase shift of 60 Carrington rotations, the second peaks had a shift of 92 rotations. These shifts corresponded to correlation coefficients of 0.824 and 0.795 respectively. For the first pair of peaks, this is a slight decrease when compared to helicity ($\Delta P = 0.06$), and a negligible change in the second case ($\Delta P = 0.045$).

Visually, the helicity-flux cycles appear to have a better fit than the polar field (with the exception of the third pair of cycles). It is, however, difficult to quantify this. We perform the same integration procedure as performed on the magnetic-helicity flux in Tables 1 and 5. Table 6 shows the results of the integration process. The results are more consistent than the aforementioned tables, with the ratio fluctuating close to $65\% $. We note that at the time of writing, the final sunspot cycle was incomplete, meaning that its integrated value is likely to increase.

**Table 6:** Integrated polar field and helicity flux ratios with integrated sunspot number.

Peak pair	Polar field integration ratio	Helicity flux integration ratio
1	68%	94%
2	77%	81%
3	52%	66%

The ratios are consistently below those of Table 1, which have been included in the rightmost column. This indicates a larger disparity between the strength and structure of the polar-field cycles, *versus* that of sunspots. The structural comments made earlier continue to apply, which is expected, given the presence of $B_{n}$ in the expression for the magnetic-helicity flux.

## Reconstructed Magnetic Field Harmonics

There have been attempts to “recreate” measurements of the large-scale solar magnetic field using a variety of proxies. One example of this is described by Makarov and Tlatov (2000), who used H$\alpha $ maps to calculate a spherical-harmonic decomposition of the said field, up to degree $\ell = 10$. This technique is broadly described by Makarov and Sivaraman (1989). The authors have generously provided their decomposition data over the period 1958 – 2015, covering Carrington rotations 1400 – 2161. This period contains two additional solar cycles that are not available in the Wilcox data.

We must, however, take care when using reconstruction data due to possible inaccuracies, and we therefore suggest that any conclusions drawn from this work are taken as secondary to that in the previous sections.

Markarov and Tlatov have also provided data covering Rotations 800 – 1400 (1913 – 1958), although they have made it clear that this second data set is more likely to be inaccurate than the more recent reconstructions. We therefore split our analysis into two sections, each dealing with one of the two periods.

### 1958 – 2015

**Figure 17:**
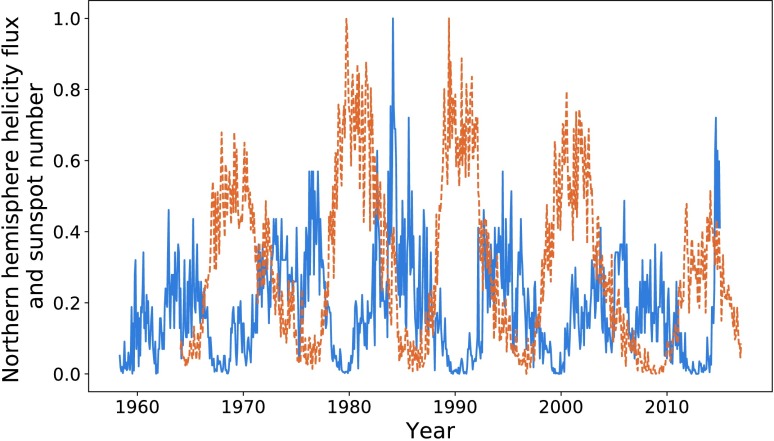
Northern hemisphere helicity flow (*blue*) and sunspot number (*dashed orange*).

**Figure 18:**
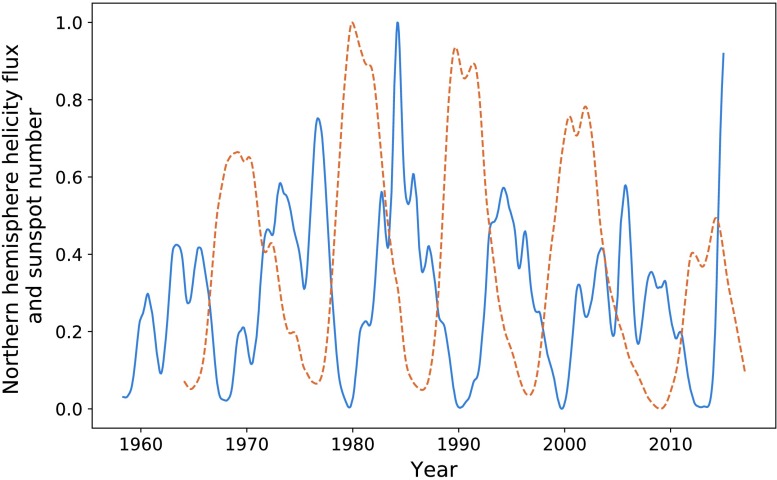
Smoothed helicity flux (*blue*) and smoothed sunspot number (*dashed orange*).

In Figure 17, we plot the helicity flux through the northern hemisphere on the same axes as sunspot number. Both data sets continue to have a cadence equivalent to one Carrington rotation. The helicity-flux data are noisier than that provided by the Wilcox Solar Observatory. This is likely due to its nature as an indirect measurement. Applying the KS as described earlier gives the data sets shown in Figure 18. The cycles appear much more correlated when smoothed, particularly in terms of their amplitude. Maximising the correlation of the two data sets over their entire range gave $P = 0.72$ with a phase shift of 84 Carrington rotations for the smoothed data, and $P = 0.56$ over a 87 Carrington-rotation shift for the unprocessed data (again over the whole data set). The difference in the phase shift is likely due to the noise, not any underlying change in structure.

**Figure 19:**
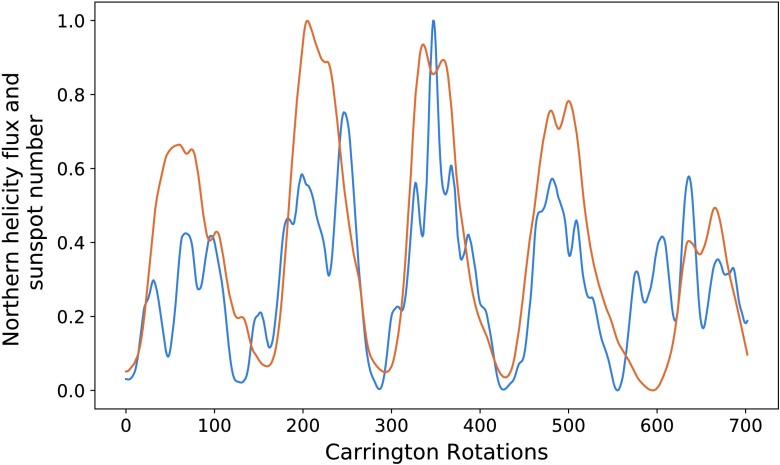
Shifted helicity flux (*blue*) compared with sunspot number (*orange*).

Maximising the correlation between each pair of peaks individually gives a small range of phase shifts: $85, 84, 81, 84 $, and 87 Carrington rotations, respectively. This corresponds to a range of 0.44 years. The optimisation of the entire data set is close enough to these values that we decide to use a mean of these values: $84~\mbox{CR}$. The result of shifting our entire helicity flux data set forwards by this amount is shown in Figure 19. We see a fairly strong correlation in terms of structure, albeit weaker than that of the previous sections. Notably, the final cycle is quite well correlated, which is where the Wilcox data appeared to “fail”. This is discussed further in the coming sections.

#### Polar Field

**Figure 20:**
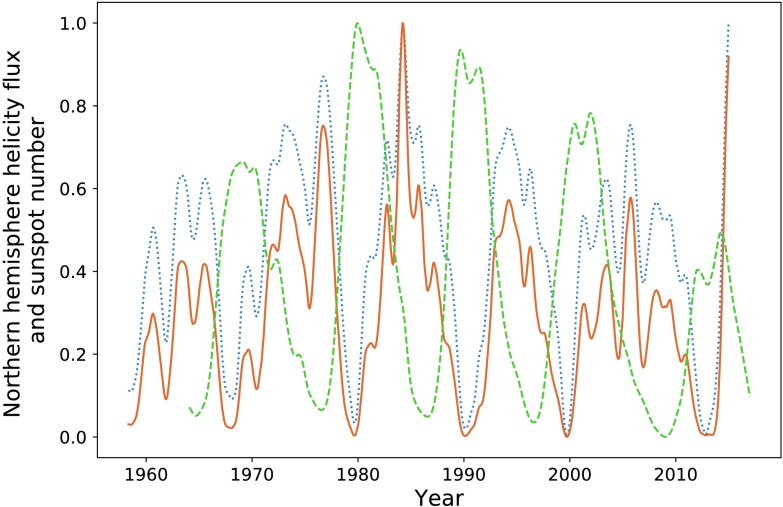
Helicity flux (*orange*) and polar field (*blue*) in the northern hemisphere compared with sunspot number (*green*).

In Figure 20, we plot the polar field, the helicity flux, and the sunspot number, all of which have been smoothed. In Table 7, we perform the same integration procedure as described during earlier analysis, and then list the results. The integration procedure has been performed on the unprocessed data. For the reconstructed data, Figure 20 indicates that the polar field instead is a (albeit slightly) superior precursor to the following solar cycle, except in the case of the final cycle. This is almost a direct contradiction of the results of the previous section. This is reflected in the table of integration ratios, where we see helicity performing much more poorly than the polar field. However, we note that data here fluctuated quite chaotically on short time scales, which has skewed our integration measure. Errors when integrating over a helicity cycle were seen to be as high as $13\%$. The polar field is taken as an average over a 15-degree latitude cap and would therefore be less subject to fast fluctuations.

**Table 7:** Integrated polar-field and helicity-flux integration ratios.

Peak pair	Helicity flux ratio (smooth)	Polar field ratio (smooth)
1	39.60% (51.89%)	96.20% (90.20%)
2	59.57% (80.69%)	97.69% (83.40%)
3	63.44% (83.57%)	81.98% (83.66%)
4	46.30% (60.23%)	99.40% (94.86%)

The advantages of smoothing are shown in the bracketed values, making the most notable difference for the helicity-flux ratio. For the two central cycles, we note that smoothing brings the ratios of the polar field and helicity flux to almost equal values.

#### Hemispherical Sunspot Number

**Figure 21:**
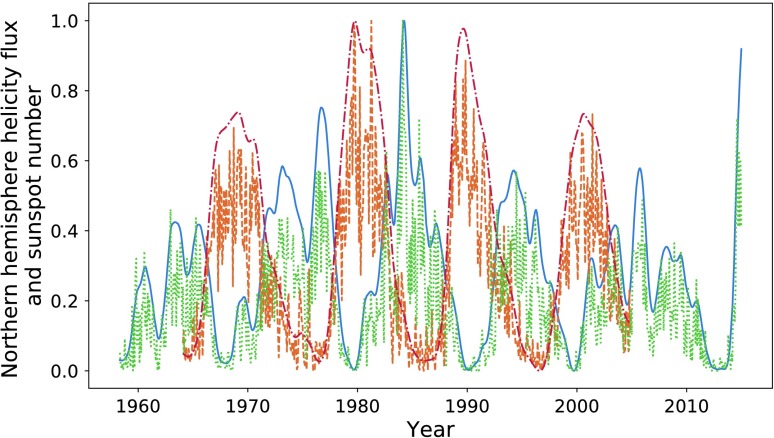
Helicity flux raw (*dotted green*) and smoothed (*blue*), compared with raw (*dashed orange*) sunspot number and smoothed (*dash–dot red*).

In this subsection we compare the reconstructed helicity flux with the reconstructed hemispherical sunspot number. We must note that we are comparing two reconstructed data sets, meaning that any inaccuracies are likely to be compounded. Figure 21 shows sunspot number compared with helicity flux, both taken strictly from the northern hemisphere. We include both the raw and the smoothed data sets, to give an indication of the magnitude of difference between them.

Table 8 shows the integration ratios for helicity and sunspots in the northern hemisphere. These have slight improvements for the smoothed data sets, and noticeable improvements when we use the “raw” data. We believe, given the chaotic nature of the reconstructed data, that the smoothed data will give a better representation, as long as we smooth both sunspot number and helicity. The median of the helicity-integration ratios when we took sunspot number over the whole disk was $69.1\%$, whilst when we split the data by hemisphere we get a mean of $77\%$: an improvement of $10\%$.

**Table 8:** Northern integrated sunspot number and helicity-flux integration ratios using hemispherical splitting.

Peak pair	Helicity flux (smooth)	Sunspot number (smooth)	Integration ratio (smooth)
1	18.32 (29.99)	41.90 (59.17)	43.7% (50.6%)
2	32.80 (54.21)	42.29 (61.66)	77.6% (88.0%)
3	30.91 (51.29)	38.10 (54.53)	81.1% (94.1%)
4	21.55 (35.40)	31.56 (45.28)	68.3% (78.2%)

#### Comparisons with Wilcox Data

In this subsection, we compare the outputs of the harmonics in the reconstructed data with the outputs produced by the Wilcox data sets. This will give a measure of the accuracy of the reconstructed data, indicating how firmly we can make conclusions from the results that it provides.

**Figure 22:**
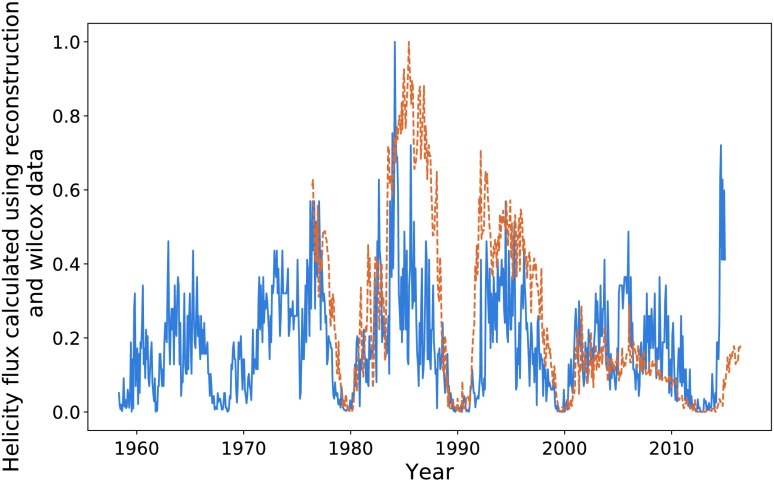
Helicity flux through the northern hemisphere calculated using Wilcox (*dashed orange*) and reconstructed spherical harmonics (*blue*).

In Figure 22, we plot the helicity flux through the northern hemisphere using both Wilcox data and the reconstructed data. The relationship between the two sets of data for was almost identical in the southern hemisphere, and we thus choose to analyse only the North.

There is a strong correlation between the two sets of helicity flux, indicating a good level of accuracy in the reconstructed data. Notable differences include the amplitude of the final observed cycle, and the “smoothness” of the cycles, particularly notable during 1980 – 1990. These differences are mostly removed using the KS process described in earlier sections. These differences in structure are what likely caused the integration procedure of the previous section to be skewed, and they are strongest during the aforementioned cycle.

Again, the final cycle is the most troublesome, giving the largest difference between the two data sets. The length of the cycles is the same for the two sets, but the amplitude is notably (approximately two times) larger in the reconstructed data. The cycle 1980 – 1990 has the largest amplitude of both sets of data, meaning that they share a common normalisation point. This means that we cannot assume that the amplitude difference is due to normalisation issues. The amplitude differences could instead be due to either an issue with the H$\alpha $ maps used to determine the magnetic harmonics, or some underlying physical mechanism linked to the extended solar minima experienced during this period. More likely is that the reconstruction technique is simply not as accurate as real-time data taken by the Wilcox Solar Observatory, and they should thus be considered as less meaningful.

**Figure 23:**
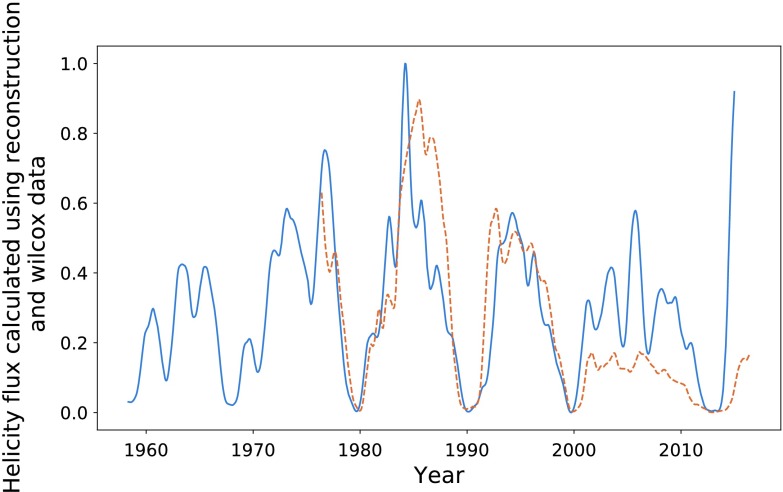
Helicity flux through the northern hemisphere calculated using Wilcox data (*dashed orange*) and reconstructed spherical harmonics (*blue*), all smoothed.

Figure 23 gives a comparison of the two smoothed data sets. Here, we see that the structures are quite similar, but we continue to observe the amplitude differences for the final cycle.

The reconstructed data also has a different relationship with its polar field than that of the Wilcox data. For Wilcox, we see an increasing cycle-amplitude difference between the two quantities with decreasing cycle strength. In the reconstructed data, however, the difference between helicity flux and polar field is fairly constant from cycle to cycle.

The exact reasons for the differences described are not known, but they are worthy of further investigation, being of particular importance for the helicity cycles of 1980 – 1990 and 2000 – 2012.

### 1913 – 2015

**Figure 24:**
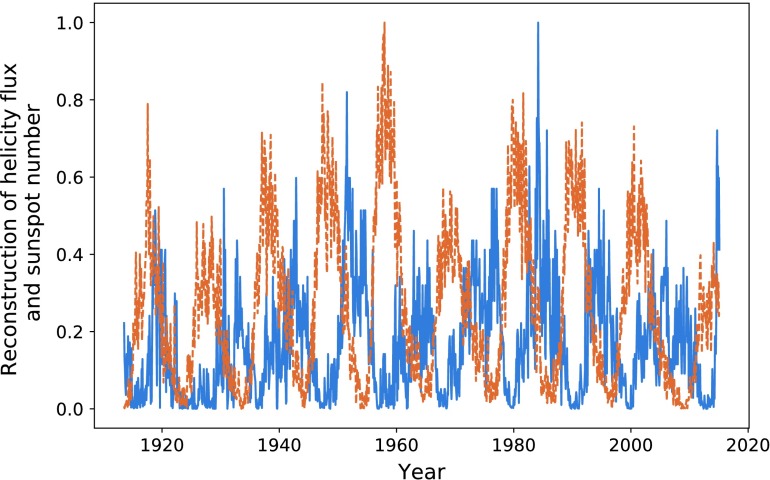
Helicity flux (*blue*) through the northern hemisphere and sunspot number (*dashed orange*).

**Figure 25:**
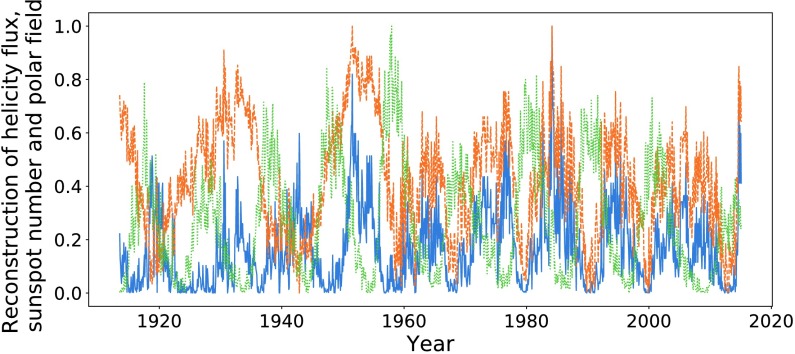
Helicity flux (*blue*), polar field (*dashed orange*) in the northern hemisphere, and sunspot number (*dotted green*).

In Figures 24 and 25 we plot the reconstructed helicity flux and polar field compared with sunspot number from 1913 onwards. Although there is a good correlation between helicity and sunspot number for dates preceding 1958, we note that the polar field of Figure 25 exhibits conjoined cycles, indicating inaccuracies in their data processing (as was indicated when the data were provided). We therefore do not perform further data analysis on this period. We note only that there continues to be a fairly noticeable visual correlation between the helicity flux and sunspot number.

## Predicting Solar Cycle 25

**Figure 26:**
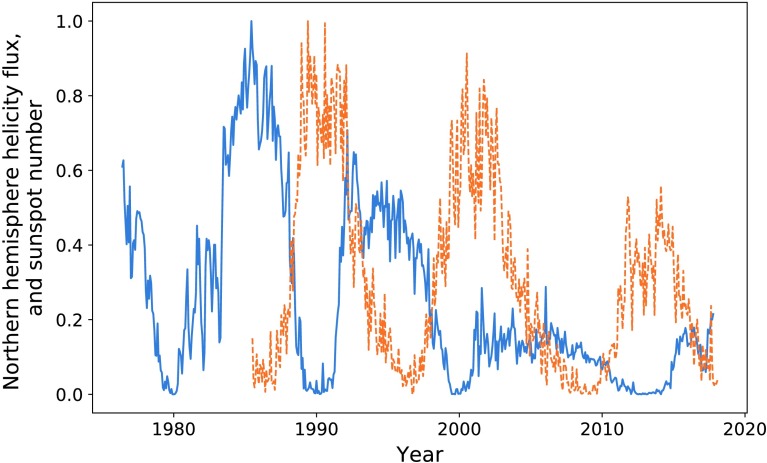
Helicity flux (*blue*) through the northern hemisphere against sunspot number (*dashed orange*).

In this section we make a brief prediction of the upcoming Solar Cycle 25. Figure 26 gives the northern hemispherical helicity flow, and total sunspot number, up to 2018 (making it more recent than other data used in this article).

We are unfortunately not at a point where we can be sure that the helicity flux has reached its peak, making the accuracy of any prediction lower than we would like.

Over the course of this article we have demonstrated statistically that the helicity-flux cycle is a good predictor of the following activity cycle. With this in mind, based on the assumption that Cycle 25’s preceding helicity-flux cycle has reached its maxima, we predict that the amplitude of Cycle 25 will be only slightly higher than that of Cycle 24.

Using our three existing cycles, we estimate that Cycle 25 will have an amplitude of 117 (given as the maximum value of sunspots per Carrington rotation over a cycle), $50 \%$ of that of Cycle 22, calculated by regression analysis on the amplitudes. This is quite similar to that of Cycle 24’s amplitude of 104. With only three data points, the standard statistical error was found to be quite un-realistic, and we therefore do not include it here.

Previous sections have also indicated that the helicity flux as a prediction mechanism is best when we compare the areas under curves. At the time of writing, the current helicity-flux cycle has an area of 5.63 CR. If we again assume that the helicity-flux cycle has reached its halfway point, this would give us a total area of ${\approx}\, 11.26$ CR.

Performing regression analysis on the areas, as an indication of the overall strength of the cycle (as in Table 1) gives a predicted normalised area of 24.48 CR for Cycle 25. This is again approximately equivalent to the area of Cycle 24 (26.50 CR). Errors have once again been neglected.

Gopalswamy *et al.* (2018) similarly found that Cycle 25 will be akin to Cycle 24.

## Conclusion

We have completed a fairly comprehensive statistical analysis of the hypothesis that helicity flux during solar minima can be used to predict the strength of the following solar maxima. We found a strong indication of causation between the two sets of data. This was most noticeable when we performed our analysis on a hemispherical basis, where we saw ratios consistently outperforming those calculated over the entire solar disc. In some cases, this ratio was more than doubled ($41.1\%$ to $93.2\%$) when using helicity flux as opposed to polar field. Notably, the strength of the polar field as a precursor seemed to decrease when we looked at individual hemispheres. This result indicates that future attempts to predict the strength of solar cycles should use a hemispherical model. In particular, the relationship appears stronger than that offered by the polar field, which is currently the most popular precursor indicator of solar activity. The only advantage that we found when using polar field was that it seemed to occasionally excel in terms of a more-exact amplitude prediction. However, this only occurred when the analysis was performed over the entire disc, rather than with respect to hemispheres. The helicity flux outperforming the polar field indicates that magnetic-field activity in regions beyond the polar cap is important for the progression of the solar dynamo into its next maxima.

In an attempt to strengthen our result, we obtained reconstructed data, but this was found to imply that the polar field was the stronger indicator, even if helicity flux still offered a high correlation. However, these data were found to be inaccurate in some places, and highly chaotic. Some underlying structure of magnetic cycles appeared to be absent. The reconstruction then, with the necessary amount of trepidation, does also indicate a causal relationship between helicity flux and sunspot number.

In conclusion, we believe that we have demonstrated the statistical link between these two physical quantities, using two different data sets over a period covering approximately 50 years.

Additionally, we made a speculative guess as to the amplitude of the forthcoming solar cycle, Cycle 25, which we believe would be approximately the same (perhaps slightly greater) amplitude and strength as Cycle 24.
